# Unraveling the triglyceride-glucose index: a key predictor of liver fat content and the amplifying role of BMI: evidence from a large physical examination data

**DOI:** 10.3389/fendo.2025.1555300

**Published:** 2025-04-25

**Authors:** Su-Juan Liu, Jin-Hui Duan, Yang-Yang Chen, Shi-Li Gu, Yu-Hua He, Ming-Mei Xue, Jun-Yan Yue

**Affiliations:** Department of Radiology, The First Affiliated Hospital of Xinxiang Medical University, Xinxiang, China

**Keywords:** triglyceride-glucose index, liver fat content, body mass index, nonlinear relationship, Chinese

## Abstract

**Background:**

The triglyceride-glucose (TyG) index is associated with the severity of metabolic-associated fatty liver disease (MASLD), but its link to liver fat content is not fully understood. This study investigates the relationship between the TyG index and liver fat content and explores the role of body mass index (BMI) as a mediator.

**Methods:**

This cross-sectional study analyzed data from 12,750 participants who underwent health screenings at the first affiliated hospital of Xinxiang Medical University between January 2018 and December 2023. The TyG index, derived as Ln [triglycerides (mg/dl) * fasting plasma glucose (mg/dl)/2], was the independent variable, while liver fat content, measured by quantitative computed tomography (QCT), was the dependent variable. Participants were grouped into tertiles based on their TyG index. Univariate and multivariate analyses, smooth curve fitting (generalized additive models), threshold effect analysis, and subgroup analyses were used to assess the TyG-liver fat content relationship. BMI’s mediating effect was also examined.

**Results:**

Liver fat content increased steadily across TyG index tertiles. After adjusting for confounders, the TyG index remained independently associated with liver fat content [β = 1.42, 95% CI: 1.26-1.57]. Participants in the highest TyG tertile (T3) had a 1.58-fold higher liver fat content compared to those in the lowest tertile (T1) (95% CI: 1.37-1.80, *P*<0.001). A generalized additive model showed a nonlinear relationship between TyG index and liver fat content. When the TyG index ≤ 7.39, liver fat content increased gradually (β = 0.74, 95% CI: 0.50-0.99, *P*<0.001). Beyond this threshold, liver fat content rose sharply (β = 2.19, 95% CI: 1.92-2.46, *P*<0.001). Subgroup analysis indicated that the association between TyG index and liver fat content was stronger at higher BMI levels (*P* for interaction < 0.001). Mediation analysis revealed that BMI accounted for 26.68% of the observed effect.

**Conclusion:**

The TyG index is positively associated with liver fat content in a nonlinear manner, with BMI amplifying this effect. These results suggest that the TyG index may be a useful marker for predicting liver fat content, and managing weight could help slow the progression of MASLD.

## Introduction

Hepatic steatosis is the abnormal buildup of triglycerides in the liver, which can progress to cirrhosis, fibrosis, hepatocellular carcinoma, and ultimately liver failure (1, 2). Metabolic-associated steatotic liver disease (MASLD) is the updated term for liver disease linked to metabolic syndrome, occurring when more than 5% of liver cells show fat accumulation (3). As obesity rates rise globally, MASLD has become more prevalent, now affecting over one-third of adults worldwide (4). In China, the prevalence has reached 29.6% in the past two decades, placing a heavy burden on healthcare systems (5). Fortunately, hepatic steatosis is reversible in its early stages and reducing fat accumulation can mitigate liver damage (6). Thus, finding accurate, reliable, and accessible biomarkers is essential for early prevention and treatment of MASLD.

The triglyceride-glucose (TyG) index is a cost-effective and widely accessible marker, proven to accurately identify metabolic syndrome (7, 8), including cardiovascular disease (9), insulin resistance (10), and obstructive sleep apnea (11). Given that fat accumulation and insulin resistance are closely linked to MASLD development (12), studies have investigated the TyG index’s relationship with MASLD, confirming its potential as a predictor of MASLD severity (12–14). However, MASLD severity is typically assessed through imaging, which is subject to the physician’s interpretation, raising concerns about consistency and reliability. Additionally, traditional diagnostic methods often fail to accurately compare the progression of the disease. While some studies have examined the relationship between the TyG index and liver fat content, measured via liver biopsy (12) or MRI (15), these studies often suffer from small sample sizes, limiting the generalizability of their findings. Thus, it is crucial to explore the quantitative relationship between the TyG index and liver fat content in a large community population and assess how obesity measures, like body mass index (BMI), mediate this relationship.

This study aims to analyze the relationship between the TyG index and liver fat content in 12,750 participants who underwent health screenings between January 2018 and December 2023, while also evaluating BMI’s mediating role in this association. Our findings could provide valuable insights for early MASLD risk assessment and intervention strategies.

## Materials and methods

### Subjects

This retrospective cohort study followed the guidelines of the Declaration of Helsinki and was approved by the Ethics Committee of the first affiliated hospital of Xinxiang Medical University (Approval Code: EC-024-599). Informed consent was waived as all participants’ personal data were anonymized, ensuring no individual could be identified.

Medical records of adults who underwent health examinations at the first affiliated hospital of Xinxiang Medical University between January 2018 and December 2023 were retrospectively analyzed. The inclusion criteria were: (1) participants who had low-dose chest CT scans that included liver fat content assessment, (2) aged 20 to 80 years, and (3) complete demographic and questionnaire data. Exclusion criteria included participants with a history or presence of cancer, severe liver disease (e.g., cirrhosis, hepatitis), severe kidney disease, metabolic disorders (e.g., primary aldosteronism, pheochromocytoma, Cushing’s syndrome), severe cardiovascular disease, pregnant or breastfeeding women, individuals with mental health conditions or mobility issues, as well as those without fasting blood glucose (FBG) or lipid profile data, or those with extreme values in these tests, were excluded from the analysis.

**Figure 1:**
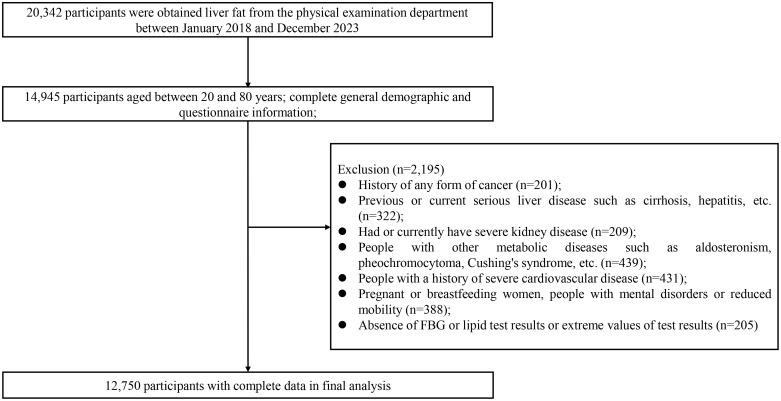
Study flowchart.

Initially, 20,342 participants were considered. After applying the exclusion criteria, 12,750 participants were included in the final analysis, with 7,592 excluded. As shown in the participant selection flowchart (**Figure 1**), individuals with absence of FBG or lipid test results (n=205) were among those excluded, ensuring that all included participants had complete data for all laboratory measurements and liver fat content. General demographic data, medical history, and medication use were collected through face-to-face interviews conducted by trained researchers. It should be noted that this study was conducted in a large health examination center where most participants’ check-up packages were sponsored by their employers as standard benefit programs, contributing to the high completeness of the laboratory and clinical data.

### Definitions of variables

The TyG index, used as the independent variable, was calculated using the formula (7): Ln [triglycerides (mg/dl) * fasting blood glucose (mg/dl)/2]. Liver fat content, assessed by quantitative CT (QCT) during health examinations, served as the dependent variable. BMI was calculated as weight (kg) divided by height squared (m²) and categorized according to Chinese standards (16) into normal weight (BMI < 24 kg/m²), overweight (24 kg/m² ≤ BMI <28 kg/m²), and obesity (BMI ≥ 28 kg/m²). Hypertension was defined as systolic blood pressure (SBP) ≥ 140 mmHg, diastolic blood pressure (DBP) ≥ 90 mmHg on two consecutive readings, self-reported hypertension, use of antihypertensive medication, or receiving antihypertensive treatment (17). Current smoking status was determined by participants’ self-reports. Current drinking was defined as consuming at least one alcoholic beverage per week within the 12 months prior to the health examination.

### Laboratory measurements

All researchers received standardized training to maintain objectivity and accuracy. Before the examinations, standardized questionnaires were used to collect key information from participants, including histories of endocrine disorders, liver and kidney diseases, cancers, lipid-lowering medications use, and anti-hyperglycemic drugs use. Once the questionnaires were completed, the researchers organized, reviewed, and verified the data. Any errors or missing information were confirmed in person or by phone.

Fasting venous blood samples were drawn from participants at 8 a.m. following a 12-hour fast. These samples were analyzed for total protein (TP), total bilirubin (TB), alanine aminotransferase (ALT), aspartate aminotransferase (AST), creatinine (Cre), uric acid (UA), FBG, total cholesterol (TC), low-density lipoprotein cholesterol (LDL-C), triglycerides (TG), and high-density lipoprotein cholesterol (HDL-C). Blood glucose was measured with the Beckman Coulter AU 5800 automated biochemical analyzer (Beckman Coulter Inc., Brea, CA, USA). Other biochemical parameters were assessed according to standard laboratory protocols.

### Liver fat level measurement

Liver fat was measured using data from low-dose chest CT scans, a routine test performed to assess pulmonary lesions during health check-ups. The scan covered the entire liver, minimizing unnecessary radiation exposure. All participants were scanned with the same CT machine, calibrated weekly using a phantom to ensure consistent data quality. Following the scans, radiologists trained in using the QCT Pro 6.1 Tissue Measurement application from Mindways software measured liver fat. This software directly measures liver fat in regions of interest (ROI) in the liver parenchyma, utilizing Hounsfield units and data from the calibration phantom. Measurements were taken from the largest cross-section of the liver, near the entry point of the right portal vein branch. ROIs were placed in three segments of the liver: the left lobe, right anterior lobe, and right posterior lobe, with one ROI in each segment. Each ROI measured approximately 300 mm² in area and 9 mm² in thickness, and the average of the three ROIs was used as the result. The average of the three ROIs was taken as the final measurement result. ROIs were carefully selected to avoid large intrahepatic vessels, bile ducts, calcifications, cysts, rib artifacts, and gas from the lungs or gastrointestinal tract, reducing partial volume artifacts and minimizing measurement error. This measurement method has been validated for use in the Chinese population (18).

### Statistical analysis

All statistical analyses were performed using R version 4.3.0 (R Foundation) and EmpowerStats (http://www.empowerstats.com, X&Y Solutions, Inc., Boston, MA). All statistical tests were two-tailed with a significance level of *P* < 0.05. For this retrospective cross-sectional study, we included all eligible participants from our health screening database. A *post-hoc* power analysis was conducted to verify the adequacy of our sample size. Based on previous studies showing correlation coefficients between the TyG index and hepatic steatosis ranging from 0.25 to 0.40, with our sample size of 12,750 participants, we achieved >99% power to detect a correlation coefficient of 0.10 or greater at a significance level of 0.05. This indicates that our sample size was more than adequate to detect even small associations between the TyG index and liver fat content.

Normality tests were performed on all datasets for continuous variables. Normally distributed variables were expressed as mean ± standard deviation, while skewed variables were reported as median (interquartile range). Group differences were assessed using t-tests or rank-sum tests. Categorical variables were presented as frequencies and percentages, with comparisons made using chi-square tests.

Univariate linear regression was conducted to evaluate the influence of different variables on liver fat content. Multivariate linear regression was then performed to examine the relationship between the TyG index and liver fat content, adjusting for covariates including sex, age, ethnicity, BMI, smoking, drinking, hypertension, TP, TB, ALT, AST, Cre, and UA. Covariates were selected by excluding those with a variance inflation factor (VIF) >10. Linear regression models were used to assess the relationship between the TyG index and liver fat content as a continuous variable. The regression coefficients (β) with 95% confidence intervals represent the change in liver fat content associated with each unit increase in the TyG index or when comparing different TyG index tertiles. Three models were built: the crude model with no adjustments; Model I, adjusting for demographic factors (sex, age, ethnicity, BMI); and Model II, adjusting for all confounders. Results from Model II were used as the basis for further analysis. The TyG index was divided into tertiles, with the lowest tertile serving as the reference, to evaluate its relationship with liver fat content. A generalized additive model (GAM) with smooth curve fitting was used to analyze the dose-response relationship between the TyG index and liver fat content. A two-stage linear regression model was also developed to explore potential nonlinear associations based on data from either side of the inflection point. The best-fitting model to describe the TyG index-liver fat content relationship was selected based on the log-likelihood ratio. Stratified analysis and interaction tests, based on Model II, were conducted to assess whether the relationship between the TyG index and liver fat content varied across subgroups. Causal mediation analysis was performed to determine the extent to which BMI mediated the relationship between the TyG index and liver fat content.

## Results

### Baseline characteristics of the participants

**Figure 2:**
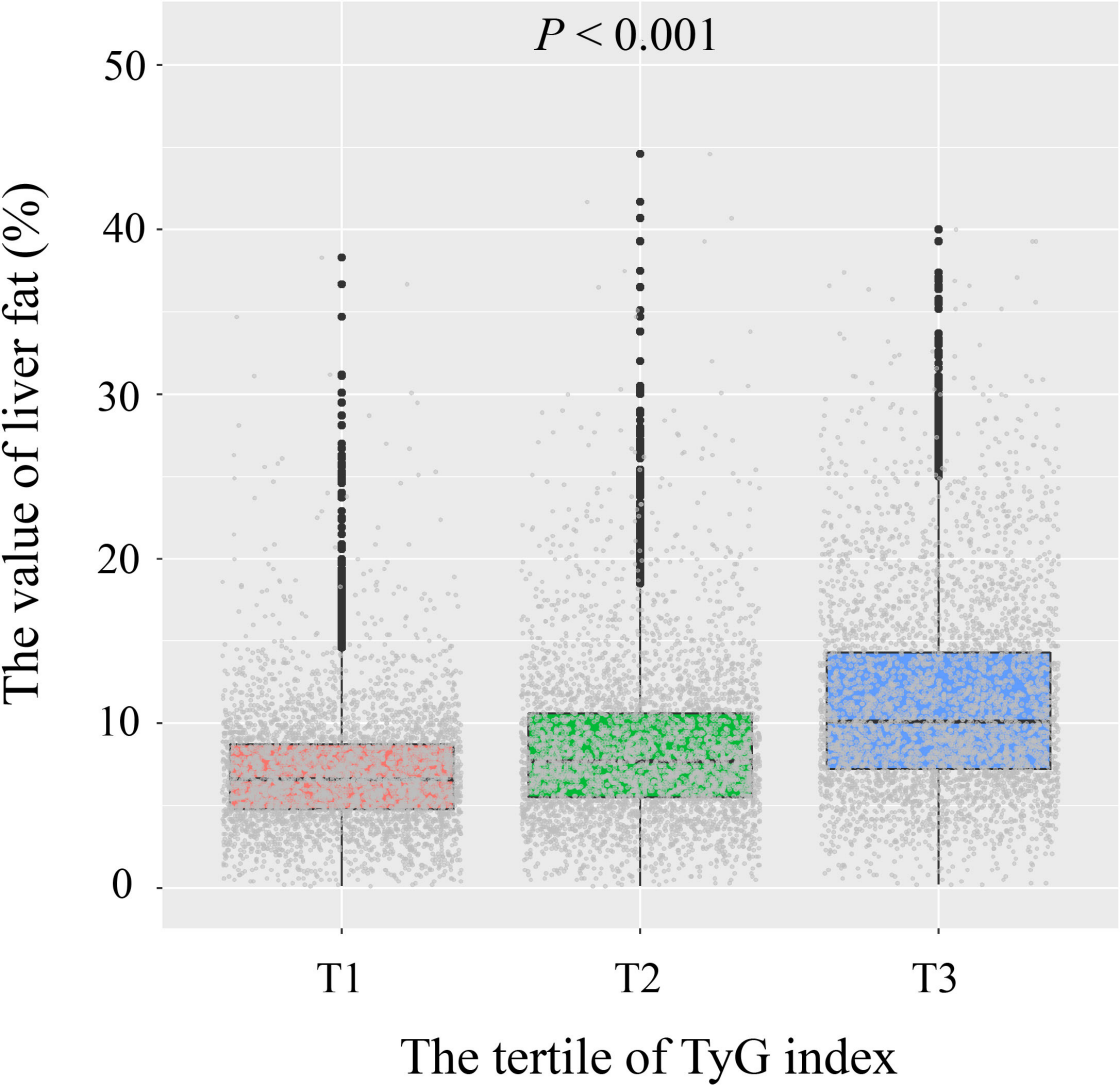
Distribution of liver fat content by tertiles of TyG index.

The study included 12,750 health examination participants, comprising 7,372 men and 5,378 women. Participants were categorized into three groups based on TyG index tertiles: T1 (5.37 ≤ TyG index < 6.89, n = 4,249), T2 (6.89 ≤ TyG index < 7.35, n = 4,251), and T3 (7.35 ≤ TyG index < 11.10, n = 4,250). **Figure 2** shows a progressive increase in liver fat content across the TyG tertiles. Participants in the T3 group (highest TyG index) were more likely to be older men with higher BMI, smoking and drinking habits, and higher blood pressure to the T1 group. They also had elevated levels of TP, ALT, AST, Cre, UA, FBG, TC, TG, LDL-C, and liver fat content (all *P* < 0.05), while HDL-C levels were lower (*P* < 0.001). No significant differences were observed in ethnic groups or TB (all *P* > 0.05), as detailed in **Table 1**.

**Table 1:** Baseline characteristics stratified by the tertile of TyG index.

TyG tertile	T1 (5.37-6.88)	T2 (6.89-7.34)	T3 (7.35-11.10)	*P*-value
N	4249	4251	4250	
Age, years	49.89 ± 11.97	51.42 ± 11.17	51.56 ± 11.12	<0.001***
< 40	2280 (53.66)	1414 (33.26)	850 (20.00)	
≥40, <60	1725 (40.60)	2264 (53.26)	2425 (57.06)	
≥60	244 (5.74)	573 (13.48)	975 (22.94)	
Sex, n (%)				<0.001***
Female	2371 (55.80)	1828 (43.00)	1179 (27.74)	
Male	1878 (44.20)	2423 (57.00)	3071 (72.26)	
Ethnic group, n (%)				0.128
Non-han	128 (3.01)	148 (3.48)	116 (2.73)	
Han	4121 (96.99)	4103 (96.52)	4134 (97.27)	
BMI, kg/m^2^	23.56 ± 2.80	25.03 ± 2.91	26.11 ± 2.90	<0.001***
< 24	2280 (53.66)	1414 (33.26)	850 (20.00)	
≥24 <28	1725 (40.60)	2264 (53.26)	2425 (57.06)	
≥28	244 (5.74)	573 (13.48)	975 (22.94)	
Current smoking, n (%)				<0.001***
No	3248 (76.44)	2940 (69.16)	2623 (61.72)	
Yes	1001 (23.56)	1311 (30.84)	1627 (38.28)	
Current drinking, n (%)				<0.001***
No	2519 (59.28)	2306 (54.25)	1961 (46.14)	
Yes	1730 (40.72)	1945 (45.75)	2289 (53.86)	
Hypertension, n (%)				<0.001***
No	3384 (79.81)	2986 (70.24)	2627 (61.81)	
Yes	865 (20.19)	1265 (29.76)	1623 (38.19)	
Medications for diabetes, n (%)				<0.001***
No	4193 (98.68)	4089 (96.19)	3520 (82.82)	
Yes	56 (1.32)	162 (3.81)	730 (17.18)	
Statins, n (%)				<0.001***
No	3759 (88.47)	3570 (83.98)	3517 (82.75)	
Yes	490 (11.53)	681 (16.02)	733 (17.25)	
TP, g/L	70.86 ± 3.96	71.57 ± 3.99	72.30 ± 4.26	<0.00***1
TB, μmol/L	12.01 ± 5.15	12.18 ± 5.17	12.18 ± 5.54	0.258
ALT, U/L	16.10 (12.40-22.00)	19.50 (14.80-27.40)	24.20 (17.80-35.20)	<0.001***
AST, U/L	19.60 (16.60-23.30)	20.40 (17.40-24.30)	21.80 (18.20-26.80)	<0.001***
Cre, μmol/L	62.91 ± 14.41	65.68 ± 13.90	67.50 ± 14.66	<0.001***
UA, μmol/L	295.97 ± 76.00	330.58 ± 81.98	364.47 ± 90.62	<0.001***
FBG, mmol/L	4.80 ± 0.54	5.12 ± 0.75	6.01 ± 1.96	<0.001***
TC, mmol/L	4.71 ± 0.85	4.98 ± 0.92	5.22 ± 1.02	<0.001***
TG, mmol/L	0.94 ± 0.21	1.52 ± 0.32	2.97 ± 1.87	<0.001***
HDL-C, mmol/L	1.49 ± 0.31	1.32 ± 0.27	1.16 ± 0.24	<0.001***
LDL-C, mmol/L	2.70 ± 0.69	3.01 ± 0.76	3.05 ± 0.81	<0.001***
TyG	6.56 ± 0.23	7.11 ± 0.13	7.82 ± 0.46	<0.001***
Liver fat, %	7.04 ± 3.56	8.52 ± 4.75	11.32 ± 5.90	<0.001***

BMI, body mass index; TP, total protein; TB, total bilirubin; ALT, alanine aminotransferase; AST, aspartate transaminase; Cre, Creatinine; UA, Uric acid; FBG, fasting plasma glucose; TC, total cholesterol; TG, triglycerides; HDL-C, high-density lipoprotein cholesterol; LDL-C, low-density lipoprotein cholesterol; TyG, triglyceride and glucose index.

All participants included in the analysis (n=12,750) had complete data for all variables presented in this table, with no missing values for any laboratory measurements or liver fat content. Except for the AST and ALT, which were expressed as medians (upper and lower quartiles), all other variables are expressed as mean ± standard deviation or counts (percentages). For age and BMI, while both descriptive statistics (mean ± standard deviation) and categorical distributions are presented, the p-values refer to the Chi-square test comparing the distribution of categorical variables across TyG index tertiles. *P*-values were calculated using one-way ANOVA for normally distributed continuous variables, Kruskal-Wallis test for non-normally distributed continuous variables (ALT and AST), and chi-square test for categorical variables. ***P < 0.001.

### Univariate analysis

Univariate linear regression analysis was conducted to evaluate the impact of traditional variables on liver fat content and to identify covariates for multivariate analysis. As presented in **Table 2**, higher age and HDL-C levels were negatively correlated with liver fat content (*P* < 0.05). Conversely, male, higher BMI, smoking, drinking, hypertension, TP, TB, ALT, AST, Cre, UA, FBG, TC, TG, LDL-C, and the TyG index were positively associated with higher liver fat content (all *P* < 0.05).

**Table 2:** Univariate linear regression analyses for liver fat content.

Variables	Statistics	β (95%CI)	*P*-value
Age, years			
< 40	1945 (15.25)	Ref	
≥40, <60	8264 (64.82)	-0.72 (-0.97, -0.46)	<0.001
≥60	2541 (19.93)	-0.57 (-0.87, -0.27)	<0.001
Sex, n (%)			
Female	5378 (42.18)	Ref	
Male	7372 (57.82)	2.44 (2.27, 2.62)	<0.001
Ethnic group, n (%)			
Non-han	392 (3.07)	Ref	
Han	12358 (96.93)	0.31 (-0.21, 0.83)	0.237
BMI, kg/m^2^			
< 24	4544 (35.64)	Ref	
≥24 <28	6414 (50.31)	2.62 (2.44, 2.80)	<0.001
≥28	1792 (14.05)	6.44 (6.19, 6.70)	<0.001
Current smoking, n (%)			
No	8811 (69.11)	Ref	
Yes	3939 (30.89)	1.75 (1.56, 1.94)	<0.001
Current drinking, n (%)			
No	6786 (53.22)	Ref	
Yes	5964 (46.78)	1.31 (1.14, 1.49)	<0.001
Hypertension, n (%)			
No	8997 (70.56)	Ref	
Yes	3753 (29.44%)	1.66 (1.47, 1.86)	<0.001
TP, g/L	71.58 ± 4.12	0.08 (0.06, 0.10)	<0.001
TB, μmol/L	12.12 ± 5.29	0.07 (0.05, 0.08)	<0.001
ALT, U/L	19.70 (14.50-28.30)	0.07 (0.07, 0.08)	<0.001
AST, U/L	20.50 (17.30-24.70)	0.08 (0.07, 0.09)	<0.001
Cre, μmol/L	65.36 ± 14.46	0.03 (0.02, 0.04)	<0.001
UA, μmol/L	330.34 ± 87.72	0.02 (0.02, 0.02)	<0.001
FBG, mmol/L	5.31 ± 1.36	0.73 (0.66, 0.79)	<0.001
TC, mmol/L	4.97 ± 0.96	0.17 (0.07, 0.26)	<0.001
TG, mmol/L	1.81 ± 0.90	1.14 (1.08, 1.20)	<0.001
HDL-C, mmol/L	1.32 ± 0.31	-5.00 (-5.29, -4.72)	<0.001
LDL-C, mmol/L	2.92 ± 0.77	0.44 (0.32, 0.56)	<0.001
TyG	7.17 ± 0.60	3.23 (3.09, 3.37)	<0.001

Abbreviations as in **Table 1**.

### Associations between the TyG index and liver fat content according to the different models

Multivariate regression analysis was performed, accounting for confounding variables, and three models were developed. In the crude linear regression model, which did not adjust for covariates, a positive correlation was observed between the TyG index and liver fat content (β = 3.23, 95% CI: 3.10 - 3.37, *P* < 0.001), as shown in **Table 3**. In Model I, after adjusting for demographic factors such as sex, age, ethnic group, and BMI, a positive correlation persisted between the TyG index and liver fat content (β = 2.04, 95% CI: 1.90 – 2.18, *P* < 0.001). In Model II, the TyG index was independently associated with liver fat content (β = 1.42, 95% CI: 1.26 – 1.57, *P* < 0.001). For each unit increase in the TyG index, liver fat content increased by 1.42 times. When divided into tertiles, after adjusting for confounders, liver fat content in the highest TyG group (T3) was 1.58 times higher than in the lowest group (T1) (*P* < 0.001).

**Table 3:** Multivariate linear regression analysis for liver fat content.

	Non-adjusted model	Adjust I model	Adjust II model
	β (95%CI) *P-*value	β (95%CI) *P-*value	β (95%CI) *P-*value
TyG	3.23 (3.10, 3.37) <0.001	2.04 (1.90, 2.18) <0.001	1.42 (1.26, 1.57) <0.001
TyG tertile			
Tertile 1	Reference	Reference	Reference
Tertile 2	1.48 (1.27, 1.68) <0.001	0.50 (0.30, 0.69) <0.001	0.18 (-0.02, 0.37) 0.075
Tertile 3	4.28 (4.07, 4.49) <0.001	2.51 (2.31, 2.72) <0.001	1.58 (1.37, 1.80) <0.001
*P* for trend	2.14 (2.04, 2.24) <0.001	1.26 (1.16, 1.36) <0.001	0.79 (0.68, 0.89) <0.001

Non-adjusted model adjusts for: None.

Adjust I model adjust for: sex, age, ethnic group, and BMI.

Adjust II model adjust for: sex, age, ethnic group, BMI, current smoking, current drinking, hypertension, TP, TB, ALT, AST, Cre, and UA. TyG, triglyceride and glucose index; CI, confidence interval.

**Figure 3:**
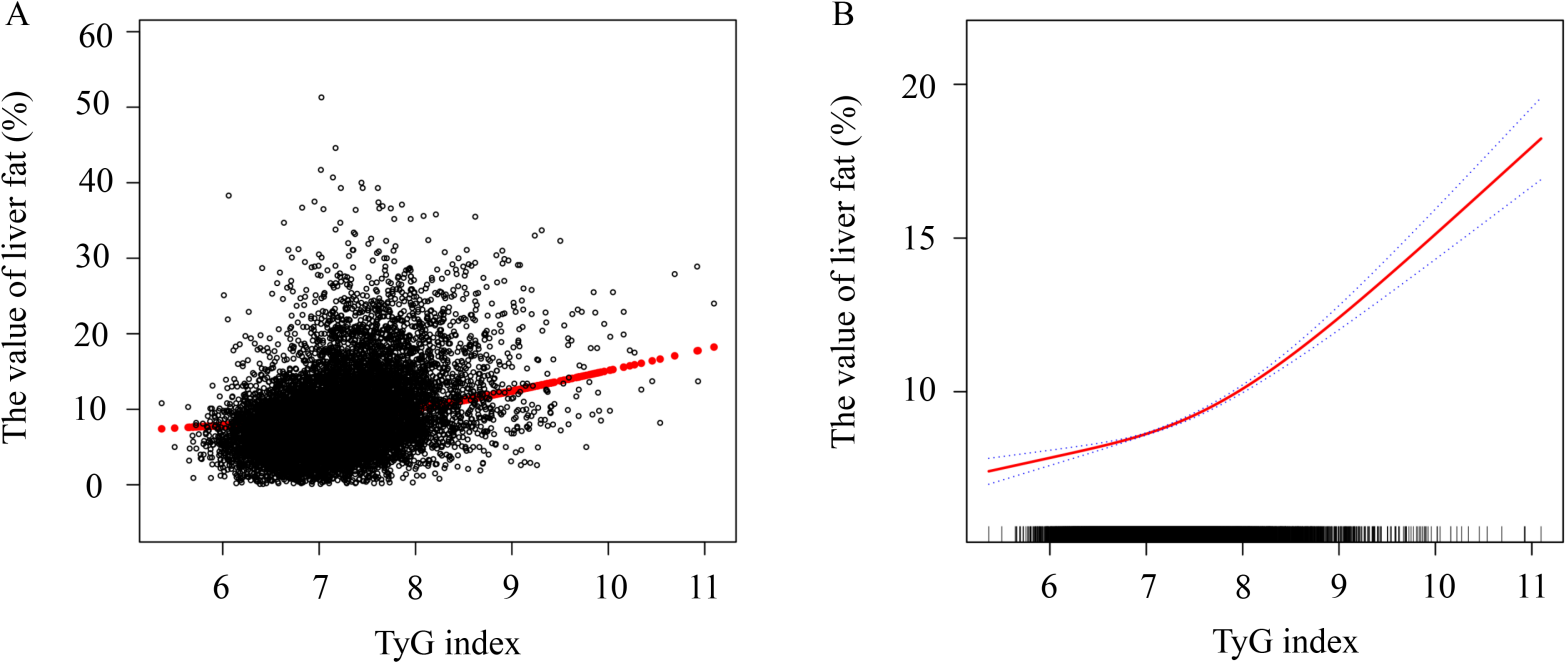
Generalized additive model with fitting smoothness for the dose–response relationship between TyG index and liver fat level. **(A)** Actual distribution of liver fat values with TyG index, black points represent one sample size, and red lines represent values after fitting. **(B)** The solid red line represents the estimate the value of liver fat, the dashed blue line represents the confidence interval of the estimate, and the short bottom line represents the sample distribution. TyG, triglyceride and glucose.

A smooth curve fitting analysis was performed to examine the nonlinear relationship between the TyG index and liver fat content. **Figure 3** illustrates a significant nonlinear relationship between the TyG index and liver fat content. Threshold effect analysis was conducted to identify the inflection point in this nonlinear relationship. After adjusting for confounders, when the TyG index < 7.39, liver fat content increased gradually with the TyG index (β = 0.74, 95% CI: 0.50 – 0.99, *P* < 0.001, **Table 4**). When the TyG index > 7.39, liver fat content increased sharply (β = 2.19, 95% CI: 1.92 – 2.46, *P* < 0.001).

**Table 4:** The result of the two-piecewise logistic regression model.

	Linear regression	Break point	< K	> K	LLR test
	β (95%CI) *P* value	(K)	β (95%CI) *P* value	β (95%CI) *P* value	*P*
**TyG**	1.42 (1.26, 1.57) <0.001^**^	7.39	0.74 (0.50, 0.99) <0.001^**^	2.19 (1.92, 2.46) <0.001^**^	< 0.001^**^

All covariates were adjusted in this model. TyG, triglyceride and glucose; CI, confidence interval.

**P < 0.001.

### Subgroup analysis

**Figure 4:**
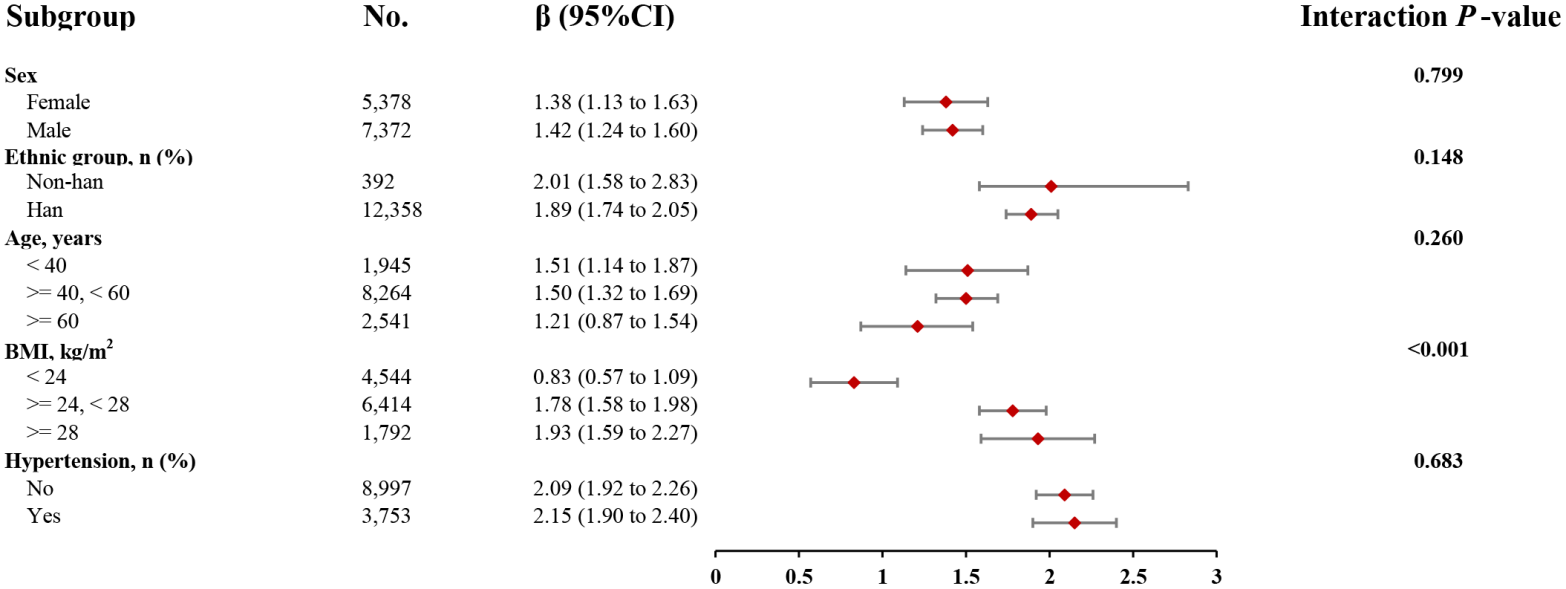
The association between TyG index of liver fat level according to different groups. Adjusted for all covariates except for this subgroup of variables. TyG, triglyceride and glucose; BMI, body mass index, CI, confidence interval.

**Figure 5:**
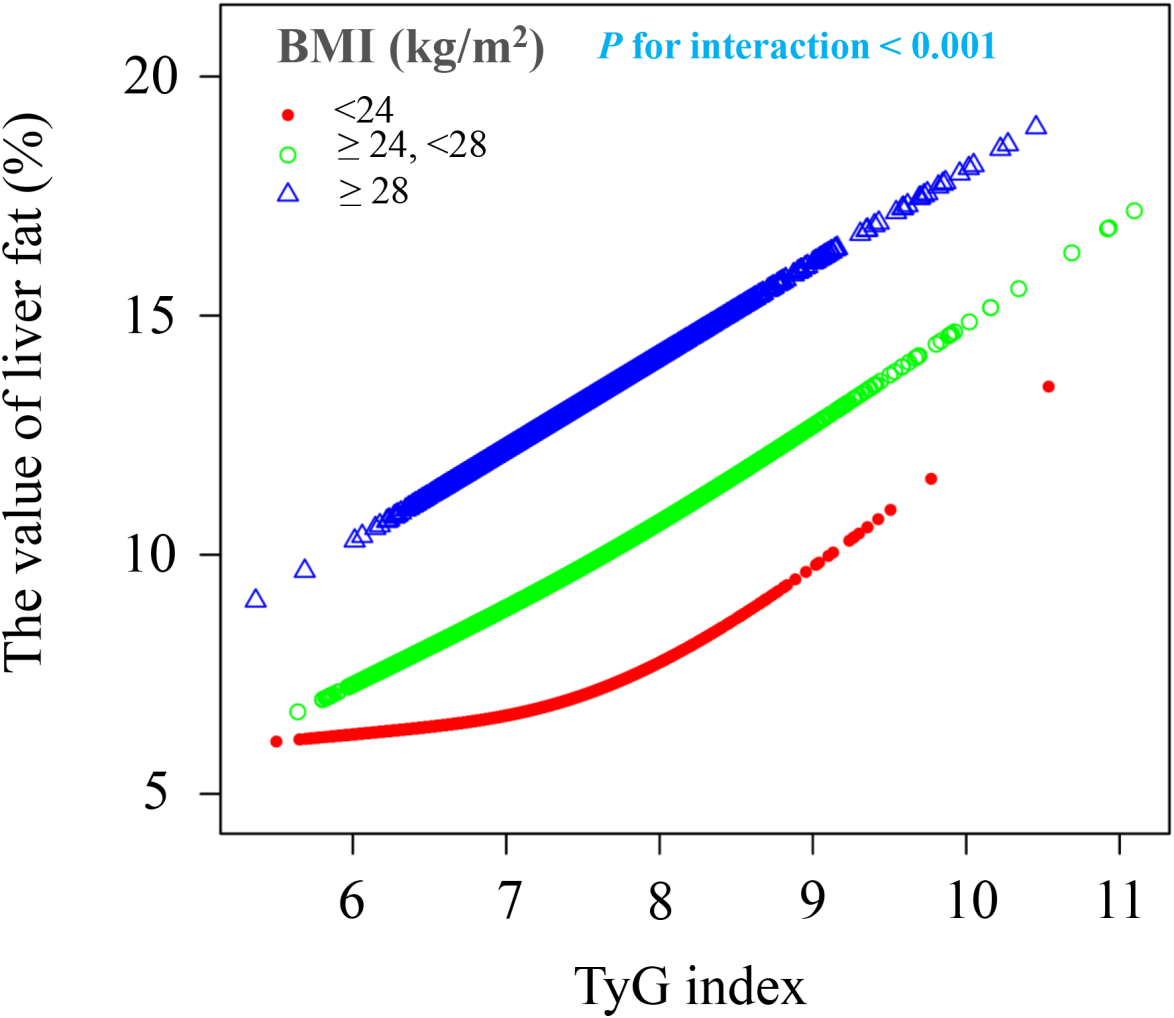
The association between TyG index of liver fat level according to different BMI groups. BMI, body mass index; TyG, triglyceride and glucose index. All covariates including sex, age, ethnic group, current smoking, current drinking, hypertension, TP, TB, ALT, AST, Cre, and UA were adjusted in this model.

Apart from BMI, the relationship between the TyG index and liver fat content was consistent across various subgroups. This independent nonlinear relationship was observed in subgroups based on age (<40 years/≥40, <60 years/≥60 years), sex (female/male), ethnicity (non-Han/Han), and hypertension status (yes/no) (interaction *P* > 0.05) (**Figure 4**). However, when stratified by BMI (<24 kg/m² or ≥24, <28 kg/m² or ≥28 kg/m²), the positive association between the TyG index and liver fat content became stronger with higher BMI (interaction *P* < 0.001). Participants with a BMI ≥28 kg/m² showed a 1.1-fold increase in liver fat content for each unit increase in the TyG index compared to those with a BMI <24 kg/m². A nonlinear positive correlation between the TyG index and liver fat content was found in the BMI <24 kg/m² group, while a linear positive correlation was seen in the 24 kg/m² ≤ BMI <28 kg/m² and BMI ≥28 kg/m² groups (**Figure 5**).

### Analysis of the mediating effect of BMI

**Figure 6:**
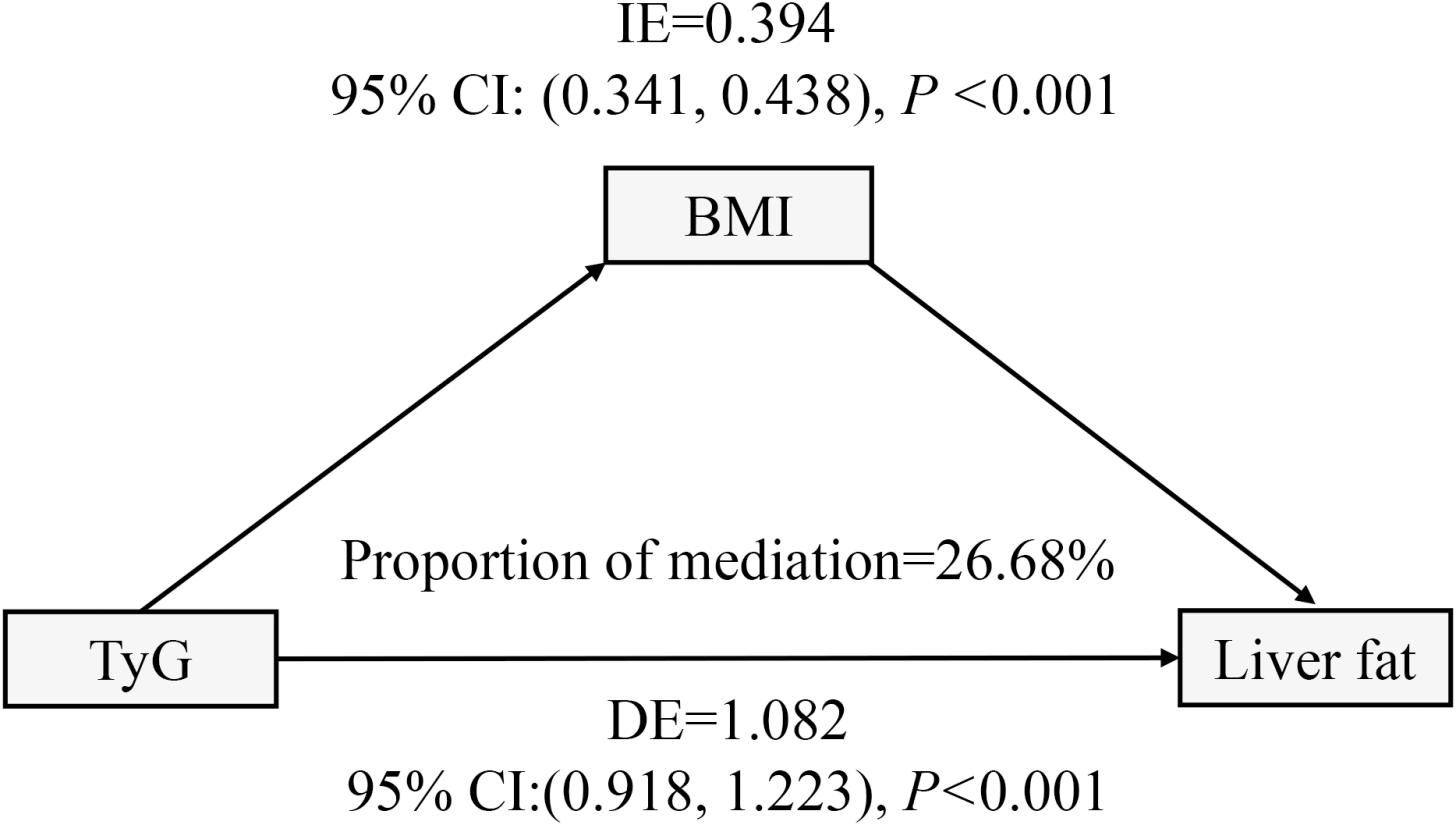
BMI in the mediation effect of TyG relationship with liver fat analysis. BMI, body mass index; TyG, triglyceride and glucose; IE, indirect effect; DE, direct effect. All covariates including sex, age, ethnic group, current smoking, current drinking, hypertension, TP, TB, ALT, AST, Cre, and UA.

Following the subgroup analysis, mediation analysis was performed to investigate the mediating effect of BMI on the relationship between the TyG index and liver fat content. As illustrated in **Figure 6**, the indirect effect of BMI as a mediator was 0.394 (95% CI: 0.341-0.438, *P* < 0.001), accounting for 26.68% of the total effect.

## Discussion

This large cross-sectional study, based on six years of liver fat content data from health examination participants, identified a non-linear positive association between the TyG index and liver fat content. After adjusting for confounding factors, liver fat content showed a marked increase when the TyG index> 7.39. This relationship held consistent across subgroups such as age, sex, ethnicity, hypertension status, diabetes medication use, and statin use, but was notably amplified by higher BMI. Our results suggest that BMI mediates around 26.68% of this effect. To our knowledge, this is the first study to quantify the relationship between the TyG index and liver fat content in such a large cohort. These findings could help primary care physicians assess MASLD risk using the TyG index and offer valuable guidance for the prevention and management of non-alcoholic fatty liver disease.

Previous studies have identified metabolic dysfunction, characterized by elevated intrahepatic triglycerides and insulin resistance, as hallmark features of MASLD (19, 20). The TyG index and related measures are independently linked to the severity of hepatic steatosis (13, 21). As a widely used indicator of metabolic dysfunction, the TyG index has gained prominence in MASLD research. Recent data from the U.S. National Health and Nutrition Examination Survey (NHANES III) and the National Death Index (NDI), covering 10,390 participants, highlight the TyG index’s strong predictive value for survival among MASLD patients (22). Additionally, a systematic review and meta-analysis of 20 studies confirmed the TyG index’s effectiveness in diagnosing and predicting new MASLD cases (23). These studies suggest that the TyG index is a valuable tool for identifying MASLD risk. Moreover, a longitudinal study of 113 metabolic syndrome patients found a significant correlation between the TyG index and liver steatosis confirmed by biopsy (12). These findings emphasize the TyG index as a key marker for assessing liver fat content.

This study, based on a large sample of health examination participants, explored the relationship between liver fat content and the TyG index. After adjusting for confounding factors, a nonlinear positive correlation was identified between the TyG index and liver fat content in the general population. When the TyG index exceeded 7.39, liver fat content increased significantly with higher TyG levels. This aligns with previous studies, which show a dose-response relationship between the TyG index and MASLD, with a greater risk of MASLD as the TyG index rises, regardless of confounding variables (24). MASLD patients commonly present with insulin resistance, disrupted glucose and lipid metabolism, and inflammation (25, 26). The TyG index is regarded as one of the simplest and most reliable markers for detecting insulin resistance and has shown significant utility in MASLD screening (27–29). However, the quantitative relationship between the TyG index and liver fat content has been insufficiently studied. Previous research suggests that liver fat accumulation is linked to glucose and lipid metabolism disorders. As liver fat content increases, insulin sensitivity decreases, leading to higher hepatic glycogen production and elevated FBG levels (30). Furthermore, liver fat accumulation triggers inflammation and stress responses, disrupting insulin signaling via pathways like c-Jun N-terminal kinase (JNK), worsening glucose dysregulation (31). Increased liver fat also promotes the synthesis of endogenous triglycerides through *de novo* lipogenesis (DNL) (20), which are packed into very-low-density lipoproteins (VLDL) and released into the bloodstream, raising plasma triglyceride (TG) levels (32, 33). The TyG index, which incorporates both FBG and TG levels, effectively reflects the severity and specificity of glucose and lipid metabolism abnormalities. Therefore, the TyG index can be a reliable predictor of liver fat content in the general population.

To confirm the stability of the relationship between the TyG index and liver fat content, a subgroup analysis was performed. The results showed that, apart from BMI, the relationship between the TyG index and liver fat content was unaffected by factors such as age, sex, ethnicity, hypertension status, diabetes medications, or statin use. Specifically, as BMI increased, the positive association between the TyG index and liver fat content became more significant. In overweight (24 kg/m² ≤ BMI < 28 kg/m²) and obese (BMI ≥ 28 kg/m²) individuals, a linear positive correlation was observed between the TyG index and liver fat content. It is well established that higher BMI is typically linked to increased body fat, especially visceral fat, which leads to the release of fatty acids into the liver (34–36). Increased BMI is also associated with chronic inflammation in adipose tissue, where inflammatory factors like tumor necrosis factor-α and interleukin-6 promote hepatic insulin resistance, leading to excessive fat buildup in the liver (37). Crucially, insulin resistance caused by elevated BMI disrupts glucose and lipid metabolism in the liver, further increasing fat accumulation and contributing to the progression of MASLD (38). Recent animal studies have shown that obese mice regulate lipid metabolism through hepatic glucuronate C5-epimerase and growth differentiation factor 15 (39). Additionally, extracellular vesicle-derived miRNAs in MASLD patients’ plasma were positively correlated with BMI, promoting fat accumulation in the liver (40). A study from China, which included 1,171 health check-up participants, found that the TyG-BMI index was more strongly associated with the severity of hepatic steatosis (41). In our study, mediation analysis revealed that BMI mediated approximately 26.68% of the effect in the relationship between the TyG index and liver fat content. These findings highlight the critical role of weight reduction in mitigating liver fat accumulation.

The key strengths of this study include the large dataset of quantitative liver fat measurements, which provided strong statistical power for the analysis. Second, this is the first study to quantitatively assess the relationship between the TyG index and liver fat content in a community-based population, confirming its potential as a predictive tool with broad applicability. Additionally, this is the first study to evaluate the quantitative role of BMI in liver fat accumulation, providing evidence to support weight management in MASLD populations. However, there are limitations to this study. First, the cross-sectional design limits our ability to establish a causal relationship between the TyG index and liver fat content. Second, we did not measure HbA1c levels, which could have provided additional information about long-term glycemic control. Future studies incorporating HbA1c measurements may offer complementary insights, particularly for evaluating glycemic variability that might not be captured by FBG alone. In addition, due to the retrospective nature of this study and limitations in the original questionnaire design, ome covariates, such as inflammatory markers (e.g., hs-CRP), drinking frequency, quantity, or patterns, were not collected due to limitations in the health screening program. This prevented us from distinguishing between moderate and heavy drinkers, which may be important as different alcohol consumption patterns could have varying effects on liver fat content and potentially influence the relationship between the TyG index and liver fat accumulation. Future studies should include more detailed assessment of alcohol consumption. Moreover, while our study focused on BMI as the primary anthropometric measure and mediator, we did not collect data on other important anthropometric parameters such as waist circumference or waist-to-height ratio, which may better reflect central obesity and visceral fat distribution. These measures might provide additional value in predicting liver fat content and understanding the mechanisms of MASLD development. Lastly, this study was conducted at a single center in China, which may limit the generalizability of the findings to other populations. These limitations point to future research opportunities for the quantitative analysis of liver fat content.

## Conclusion

This study identified a nonlinear positive association between the TyG index and liver fat content in a community population. When the TyG index > 7.39, the relationship between the TyG index and liver fat content became significantly stronger, with higher BMI further intensifying this association. Thus, the TyG index could be a useful predictive tool for liver fat content, and weight reduction may help slow MASLD progression.

## Data Availability

The raw data supporting the conclusions of this article will be made available by the authors, without undue reservation.
